# Swimming Performance and Oxygen Consumption as Non-lethal Indicators of Production Traits in Atlantic Salmon and Gilthead Seabream

**DOI:** 10.3389/fphys.2020.00759

**Published:** 2020-07-07

**Authors:** Arjan P. Palstra, Jeroen Kals, Thijs Böhm, John W. M. Bastiaansen, Hans Komen

**Affiliations:** ^1^Wageningen University & Research Animal Breeding and Genomics, Wageningen Livestock Research, Wageningen, Netherlands; ^2^Wageningen Marine Research, Wageningen University & Research, Yerseke, Netherlands; ^3^Wageningen University & Research Animal Nutrition, Wageningen Livestock Research, Wageningen, Netherlands

**Keywords:** aquaculture, selective breeding, feed conversion ratio, starvation-refeeding, swim-tunnel respirometry, metabolic rate

## Abstract

The aim of this study was to investigate swimming performance and oxygen consumption as non−lethal indicator traits of production parameters in Atlantic salmon *Salmo salar* L. and Gilthead seabream *Sparus aurata* L. A total of 34 individual fish of each species were subjected to a series of experiments: (1) a critical swimming speed (Ucrit) test in a swim-gutter, followed by (2) two starvation-refeeding periods of 42 days, and (3) swimming performance experiments coupled to respirometry in swim-tunnels. Ucrit was assessed first to test it as a predictor trait. Starvation-refeeding traits included body weight; feed conversion ratio based on dry matter; residual feed intake; average daily weight gain and loss. Swim-tunnel respirometry provided oxygen consumption in rest and while swimming at the different speeds, optimal swim speed and minimal cost of transport (COT). After experiments, fish were dissected and measured for tissue weights and body composition in terms of dry matter, ash, fat, protein and moist, and energy content. The Ucrit test design was able to provide individual Ucrit values in high throughput manner. The residual Ucrit (RUcrit) should be considered in order to remove the size dependency of swimming performance. Most importantly, RUcrit predicted filet yield in both species. The minimal COT, the oxygen consumption when swimming at Uopt, added predictive value to the seabream model for feed intake.

## Introduction

With the general aim of improving the efficiency and profitability of Atlantic salmon and Gilthead seabream farming by selective breeding, an important development would be the identification of accurate indicators for target traits that manifest late in life, such as survival, or traits that are difficult to measure, such as individual feed efficiency. Such indicator traits should preferably be measured non-lethally and in a high throughput manner in order to phenotype a multitude of individuals from different families to be able to determine heritability of the target traits and estimate breeding values.

Promising indicator traits may come from physiological phenotyping (physiotyping) by challenge tests such as a stress challenge test, immune challenge test, hypoxia challenge test or exercise challenge test (i.e., swimming performance test). Maximal and optimal swimming speeds, and routine and active metabolic rates, may correlate with traits that either target robustness (and resilience) or growth performance in relation to feed intake. Such correlations have hardly been studied in fish thus far.

The critical swimming speed Ucrit can be assessed by subjecting the fish to a standardized test where swimming speeds are incrementally increased at prescribed intervals until fish fatigue (Brett, 1964; Plaut, 2000). Ucrit characterizes the physical resilience to exhaustive exercise and could predict physiological traits such as stress coping capacity (Vandeputte et al., 2016), cardiac ability (Claireaux et al., 2005; Anttila et al., 2014) and disease resistance (Castro et al., 2013). Attempts to predict growth performance (Vandeputte et al., 2016), feed intake and filet yield by critical swimming speed are not conclusive and the predictive value of Ucrit may be species dependent.

By performing respirometry in swim-tunnels, oxygen consumption can be measured in resting or active state and at various swim speeds. Oxygen consumption can be either expressed in relation to time (i.e., mg O_2_ h^–1^) or distance (i.e., mg O_2_ m^–1^), the latter of which is referred to as cost of transport (COT). The minimal COT value represents the smallest possible oxygen consumption for covering a unit of distance. Minimal COT defines the metabolic optimum swimming speed Uopt. Aerobic metabolic rates, the energy demand per unit of time as met by the consumption of oxygen, show high variation between individuals but also significant repeatability within individuals (in seabass; Marras et al., 2010) suggesting the presence of genetic variation for this trait. As such, oxygen consumption and any of the derived parameters may correlate strongly to production traits that have a strong metabolic relation such as growth and feed intake.

In this study, the role of swimming performance and oxygen consumption parameters as non-lethal predictors of production parameters such as growth, feed intake, intestinal fat and filet yield, was assessed, using Atlantic salmon *Salmo salar* and Gilthead seabream *Sparus aurata* as example species. Selective breeding programs for Atlantic salmon are older (10–11 generations in 2016) than those for Gilthead seabream (1–5 up to 7 generations in 2016; Chabot et al., 2016; Janssen et al., 2017) where most strains have hardly been subjected to selective breeding. The most favorable response for each of the species is growth which includes an indirect positive response on feeding efficiency (Kause et al., 2006). Feeding behavior is very different between both carnivorous species as Atlantic salmon is a pursuit predator and Gilthead seabream feeds on benthic organisms. A series of experiments was executed to provide paired observations on parameters involved in critical swimming performance, starvation and refeeding, and oxygen consumption rates in rest and when swimming. The aim was to estimate phenotypic correlations as a first proxy for further research aimed at establishing genetic parameters for these traits.

## Materials and Methods

### Ethics Statement

Experiments were conducted at the Wageningen Marine Research (WMR, formerly known as IMARES) aquaculture facilities in Yerseke, Netherlands. Experimental protocols complied with the current laws of Netherlands and were approved by the animal experimental committee of Wageningen Research (DEC nr. 2014064).

### Experimental Rationale

**FIGURE 1:**
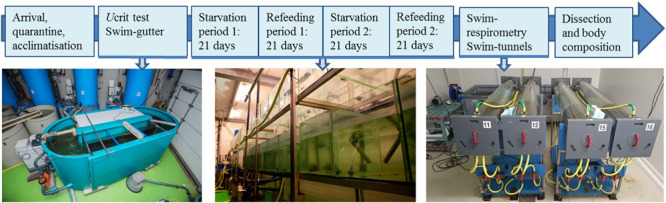
Schematic overview of the subsequent experiments.

A series of experiments was executed in order to phenotype individual fish on basis of physiological characteristics (Figure 1) consisting of two swimming trials and two starvation-refeeding periods. Firstly, a critical swimming speed (Ucrit) test was applied in a swim-gutter during which the whole population of experimental fish swam at increasing speed increments. When individual fish of the population fatigued, their critical swimming speed could be calculated. Subsequently, two starvation and refeeding experiments were executed with these fish to be able to determine weight loss, compensatory growth performance and feed conversion ratio. Finally, swimming experiments and respirometry were performed in swim-tunnels in order to determine the oxygen consumption rates, specifically the oxygen consumption in rest and during swimming at increasing speed increments to determine the COT and optimal swimming speed. The fish were then euthanized, measured and sampled for a number of parameters (described in section “dissection”). This way we were able to calculate phenotypic correlations of Ucrit, oxygen consumption in rest, optimal swim speed and minimal COT, with traits body weight, feed conversion ratio, residual feed intake, average daily weight gain and loss; organ weights and body composition.

### Experimental Fish

Atlantic salmon were obtained from a commercial salmon hatchery (Salmobreed AS, Bergen, Norway) and Gilthead seabream from a commercial seabream hatchery (Andromeda SA, Greece). After arrival, fish were acclimated for at least 14 days in a 800 L tank connected to an aquaculture recirculation system, or RAS, with a water renewal rate of 20% of the total volume per day. During acclimation, salmon were fed *ad lib* with a commercial Skretting pellet (crude protein 43%, ether extract 29%, ash 7%, 3 mm) and seabream with a commercial IRIDA pellet (crude protein 46%, ether extract 18%, ash 9.5%, 3 mm) using automatic belt feeders. For the whole period, water temperature was maintained at 14°C for salmon in freshwater and 20°C for seabream in seawater at 30 ppt. Water was aerated to ensure sufficient oxygen levels. Photoperiod was set at 17L:7D for both species.

### Experiment 1: Critical Swimming Speed (Ucrit) Test in a Swim-Gutter

After acclimation, a total number of *N* = 34 fish were introduced in a swim gutter and subjected to an Ucrit test. This procedure was repeated twice with different batches of fish to check replicability of the experiment and to have characterized back-up individuals for subsequent experimenting.

The Ucrit test was conducted in a 3,600 l oval-shaped swim gutter which was connected to a recirculating aquaculture system as described by Palstra et al. (2015; Figure 1). Maximal flow was reached at 1.2 m s^–1^.

Fish were acclimated overnight in the gutter at minimal flow to ensure sufficient water quality until the Ucrit test commenced. The Ucrit test consisted of swimming at speeds in the range 0.3 up to 1.2 m s^–1^ with increments of 0.1 m s^–1^ and swimming 20 min at each speed. The experimenter was constantly present to observe fish swimming behavior and occurrence of fatigue. When a fish fatigued, it was touching the back fence. Fatigued fish were taken out, weighed and standard length (SL) was measured, and then transferred to an aquarium for recovery. The exact time of fatigue was recorded to be able to calculate the critical swimming speed (Ucrit) according to Brett (1964) as follows:

(1) Ucrit=U-1+(t/Δ⁢t)⁢Δ⁢U

where Ucrit is the critical swimming speed in m s^–1^, (absolute Ucrit), Δt is time increment in min, ΔU is velocity increment in m s^–1^, *t* is time elapsed at final velocity in min, and U_–__1_ is the highest velocity maintained for the prescribed time period in m s^–1^.

The relation SL-Ucrit was plotted and the residual Ucrit (RUcrit) was calculated by determination of the difference in Ucrit of an individual fish and the predicted value calculated on basis of the linear regression equation: Ucrit = *SL* + *e*.

### Experiment 2: Starvation and Refeeding

Fish were tagged, weighed individually and randomly assigned to one of the 34 experimental aquaria (0.4 m^2^, 130 l), integrated in a RAS with a flow of 4.3 ± 0.6 (salmon) or 4.0 ± 0.4 (seabream) l min^–1^ tank^–1^. The daily refreshment rate was set at 10% of the total system volume of fresh water or seawater, respectively. During the experimental period the fish went through two periods of feed deprivation, which started at respectively day 0 and day 42 (Figure 1). During these two periods, fish were not fed for 21 days. Each starvation period was followed by a recovery period of 21 days (starting at day 21 and 63) (Figure 1) during which the fish were fed to satiation twice a day (9:00–10:00 and 15:00–16:00 o’clock). Individual fish were kept in their assigned aquaria during the whole experiment.

During the whole experimental period the fish were individually weighed and measured on a weekly basis to calculate weight gain / weight loss. While handling, fish were anesthetized using clove oil. Temperature, oxygen content and pH were measured daily. TAN (total ammonia nitrogen) and NO_2_ were measured only twice to check values as system load was very low with only one fish per tank and the refreshment rate set at 10%. Husbandry conditions for salmon during the whole period were: temperature 14.3 ± 0.12°C, oxygen 10.1 ± 0.5 mg l^–1^, pH 8.3 ± 0.3, TAN 0.0 ± 0.0 mg l^–1^ and NO_2_^–^ 0.008 ± 0.008 mg l^–1^. Husbandry conditions for seabream during the whole period were: temperature 19.6 ± 0.34°C, oxygen 7.5 ± 0.3 mg l^–1^, pH 8.1 ± 0.1, TAN 0.03 ± 0.06 mg l^–1^, NO_2_^–^ 0.02 ± 0.04 mg l^–1^, NO_3_^–^ 0.61 ± 0.3 mg l^–1^.

The feed intake was calculated on dry matter basis. Knowing the individual feed intake, the individual feeding efficiency and residual feed intake could be calculated. Weights were used to calculate the average daily weight gain (g d^–1^) per period per individual (ADG) during refeeding periods and the average daily weight loss (g d^–1^) per period per individual (ADL) during starvation periods. Individual feed intake in g d^–1^ was estimated as the sum of the feed eaten in the morning and the afternoon, per period by the weight of the pellets given, minus those pellets that were recovered, times the average pellet weight. Feeding efficiency per period was expressed in the feed conversion ratio on dry matter (FCR_DM_) and was calculated using: FCR_DM_ = (Feed intake × dry matter diet)/weight gain (growth). The residual feed intake RFI on dry matter basis is the difference between the estimated daily feed intake on dry matter basis times dry matter content minus the expected feed intake on dry matter basis. The coefficients to calculate the expected feed intake (on dry matter) are obtained from linear regression of the estimated daily feed intake on mid metabolic body weight and average daily weight gain as proposed by Koch et al. (1963). The expected dry matter intake is then calculated as: *Y* = β0 + β1 *X*1 + β2 *X*2 + ε, where *Y* is the expected dry matter intake, β0, β1, and β2 are the coefficients of the equation estimated from regression, *X*1 is the mid metabolic body weight (MMW), *X*2 is the average daily weight gain (ADG), and ε is the residual. The intercept of the equation was tested (using ANOVA, SPSS IBM) and when it was not significant, a new equation was fitted without the intercept. Then, the predicted feed intake of each animal was estimated using the estimated Betas equation. The actual feed intake minus the predicted feed intake corresponds to the residual feed intake. FCR_DM_, RFI, ADL, and ADG were calculated for both recovery period 1 (day 21 to 42) and period 2 (day 63 to 84) (Figure 1).

### Experiment 3: Swimming Performance and Respirometry in Swim-Tunnels

To execute swimming tests, four 127 L Blazka-type swim-tunnels (van den Thillart et al., 2004, for a detailed description; Figure 1) were used. Swim-tunnels were placed in a climate chamber maintaining air temperature at 14°C for salmon and 20°C for seabream. Each tunnel was connected to a 400 l tank filled with water, which was aerated to maintain high oxygen levels. Water from the tanks was recirculated through each tunnel using an EHEIM pump (Universal; EHEIM GmbH & Co., KG, Deizisau, Germany). The water inlet could be closed by a valve when oxygen measurements were done. A bypass with an oxygen probe in a 4-channel respirometry system (DAQ-PAC-G4; Loligo Systems Aps, Tjele, Denmark) allowed measuring total oxygen saturation of the water in percentage, which dropped due to oxygen consumption of the fish (ΔO_2_%).

One experiment with four tunnels was performed per experimental day. So per four, fish were transferred from the aquaria, anesthetized, length and weight was measured and fish were overnight acclimated in the swim-tunnels. Fish were not fed for 24 h before experimental trials commenced. Fish need to be in a post−absorptive state to measure oxygen consumption in rest (Chabot et al., 2016) and under these experimental conditions, 24 h is appropriate for salmon (Sveier et al., 1999) and seabream (Andrade et al., 1996). The experimental trial started with 1 h of oxygen measurements with fish in rest: with the propeller creating a flow of 0.1 m s^–1^ allowing the fish to move spontaneously in any direction. After 1 h, the swimming speed was set at 0.2 m s^–1^ and subsequently increased each hour to 0.4, 0.6, 0.8, and 1.0 m s^–1^, respectively. Before swimming speed was increased, tunnels were flushed for 10 min to re-establish high oxygen levels. Fish were allowed to acclimate to a newly set swimming speed for 10 min before oxygen measurements commenced. Hence, oxygen measurements were done for 40 min per swimming speed. After the swimming tests (eight experimental days times four fish), fish were transferred back to their original aquarium. The experimenter was constantly present to observe swimming behavior and to determine whether fish were benefitting from lower flow near the walls at higher speeds but such behavior was not observed. Back ground consumption (tunnels without fish) was measured but was nil in fresh tap water. The solid blocking effect was calculated (Bell and Terhune, 1970) but was negligible at this fish size in these tunnels.

From the decreasing oxygen contents when valves were closed, the oxygen consumption (MO_2_ in mg O_2_ min^–1^) could be calculated using the following formula:

(2) $$M\,O_{2}=\left(\frac{\Delta \,O_{2}\%\,\left(\text{DOmax}\times \frac{L}{100}\right)}{t}\right)$$

where DOmax is the maximum amount of oxygen dissolved in freshwater (10.29 mg O_2_ l^–1^ at a temperature of 14°C in freshwater for salmon and 9.47 mg O_2_ l^–1^ at a temperature of 20°C in seawater for seabream) and *L* is the volume of the swim tunnel and *t* the time in minutes. The COT (COT in mg O_2_ km^–1^) of the fish could then be calculated for each swimming speed.

The calculated COT was plotted against swimming speed (m s^–1^) as a polynomial U-shaped curve, from which optimal swimming speed (Uopt in m s^–1^ and SL s^–1^) and minimal COT (COTmin) could be derived (described by Palstra et al., 2008). COTmin is the point in the curve where COT is lowest and Uopt is the speed corresponding with COTmin. Residual MO_2_ (RMO_2_rest), residual COTmin (RCOTmin) and residual Uopt (RUopt) were calculated from their relations with SL as described for RUcrit.

## Dissection

After the last series of swimming exercise, all fish were euthanized using an overdose of clove oil to measure and determine carcass traits. For salmon, parameters included total length (TL, cm); body weight (BW, g); fat percentage (FAT; Distell Fat Meter); visceral fat (FATi, g); liver weight (Wliver, g); oesophagus weight (Woes, g) and length (Loes, mm); stomach weight (Wstom, g) and length (Lstom, mm); posterior intestine weight (Wpc) and length (Lpc, mm); median intestine weight (Wmedint) and length (Lmedint, mm); filet weight (Wfilet) and length (Lfilet, mm), and carcass weight (Wcarcass). Proximate composition (Ash, Fat, Moist = water content) and Prot as percentage of body weight) and energy content (Cal) expressed in MJ kg^–1^ using Bomb calorimetry of carcass were analyzed at Rikilt (Wageningen, Netherlands).

For seabream, some parameters were slightly different: viscera weight (Wviscera, g); oesophagus-stomach-posterior intestine weight (Woes-stom-pc, g); intestine weight (Wint, g) and length (Lint, mm); and body weight degutted (BWdegutted, g). Haematocrit (Hct) was determined by extracting blood from the caudal vein using a heparinized syringe and spinning it down using heparinized capillaries in a haematocrit centrifuge.

All parameters were scanned for their size correlations. When significant size correlation existed, parameters were normalized for either TL, SL, or BW depending on whether the parameter was a length or weight parameter, by calculating the index value expressed as: (parameter/TL, SL or BW) × 100. The parameters that were not correlated with size and thus not normalized were Prot and Hct. All other carcass traits were correlated with size and thus normalized and referred to as index values.

### Correlation Analyses

Residual Ucrit is a swimming parameter that may be a predictor for production traits. Indicators for production traits on basis of respirometry were RUopt; RCOTmin, and RMO_2_rest. The production traits on basis of growth performance and feeding efficiency included the response variables body weight at the start of the first refeeding period (BWstart1); average daily weight loss during the first starvation period (ADL1); feed conversion ratio on dry matter basis (FCR_DM_1); residual feed intake (RFI1); average daily growth (ADG1); and body weight at the start of the second refeeding period (BWstart2), average daily weight loss during the second starvation period (ADL2); feed conversion ratio on dry matter basis (FCR_DM_2); residual feed intake (RFI2), and average daily growth (ADG2). Carcass traits on basis of dissection included TL; BW; FAT; FW; FATi; Wliver; Woes; Loes; Wstom; Lstom; Wpc; Lpc; Wmedint; Lmedint; Wfilet; Lfilet; Wcarcass; Ash; Cal; Fat; Prot and Moist for salmon and FATi; Wviscera; Wliver; Woes-stom-pc; Wint; Lint; Wfilet; BWdegutted; Wcarcass; Ash; Cal; Fat; Prot and Moist for seabream.

Correlations between swimming performance and oxygen consumption parameters vs. growth and dissection parameters were calculated using bivariate Spearman’s Rho two-tailed correlation tests in SPSS 22 (IBM). Correlations were considered significant at *P* < 0.05.

### Prediction of Traits

The predictor variables, RUcrit, RUopt, RCOTmin, and RMO_2_rest were first tested for their individual effects on response variables, ADG, FCR_DM_, and RFI using the model:

(3) Y=u+BW⁢start+X+e

where *Y* is the trait to be predicted in period 1 or period 2, *u* is the overall mean of the trait in the specific period, BWstart is the start weight in the specific period and *X* is one of the predictor variables RUcrit, RUopt, RCOTmin, and RMO_2_rest. The covariable BWstart is included in the model to correct the response variables for the individual start weights. For FI in refeeding period 1 or 2, the covariable ADG, the average daily weight gain in the specified period, was added to the model. Models were fitted using the *lm()* function of R. The models were tested against a reduced model with the predictor variable removed using the *anova()* function in R.

Residual Ucrit was tested as predictor for carcass traits using the same model (3), where BWstart was removed from the model for those traits where it had no significant effect.

## Results

### Experiment 1: Critical Swimming

**FIGURE 2:**
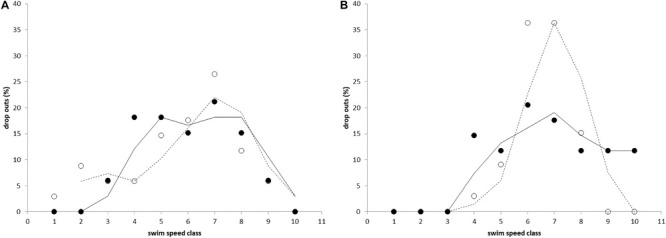
The percentage of fish that fatigued and dropped out during the critical swimming speed test for **(A)** Atlantic salmon and **(B)** Gilthead seabream. Percentage of drop outs is shown vs. swim speed classes (10 size classes of 5 cm s^–1^ in a range of 70–120 cm s^–1^) and indicates normal distributions of data. Results of both tests (filled and non-filled circles, and lines and dashed lines for the first and second test, respectively) are similar.

The developed Ucrit test proved to be very efficient in determining the individual Ucrit values for both species at this size range without causing damage or extensive discomfort. Repeats of the test for two batches of individuals showed that the distribution of results is the similar (Figure 2).

#### Atlantic Salmon

**FIGURE 3:**
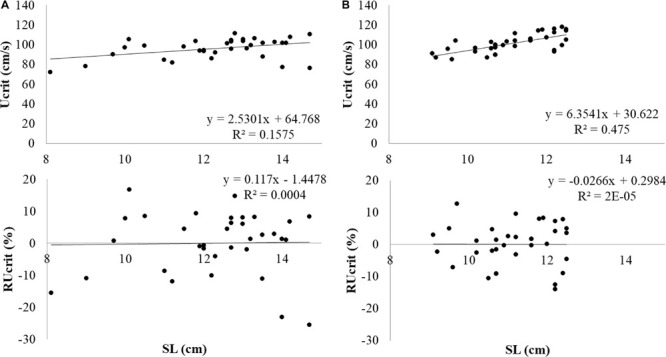
Absolute Ucrit in m s^–1^ and RUcrit in relation to SL from top to bottom in **(A)** Atlantic salmon and **(B)** Gilthead seabream.

Experimental salmon which had been subjected to the Ucrit test (*N* = 34) weighed 29.9 ± 9.9 g and SL was 12.3 ± 1.6 cm. During the test, fish were swimming in the middle at the front and near the bottom. The first fish fatigued after swimming 2 h and 2 min, after swimming 2 min at 0.9 m s^–1^. The last fish fatigued after swimming 3 h and 3 min, after swimming 3 min at 1.2 m s^–1^. Data was normally distributed. Ucrit was determined at 95.9 ± 10.3 cm s^–1^ or 7.9 ± 1.0 SL s^–1^. Critical swimming performance clearly depended on fish size (Figure 3A). When critical swimming performance is considered in absolute terms e.g., as Ucrit in cm s^–1^ vs. size as SL, larger fish swam faster. The linear relation between Ucrit and SL was *y* = 2.5301*x*+64.768 with *x* = SL (cm) and *y* = Ucrit in cm s^–1^.

#### Gilthead Seabream

Experimental seabream which had been subjected to the Ucrit test (*N* = 34) weighed 36.5 ± 9.4 g and SL was 11.2 ± 1.0 cm. Seabream were also swimming in the middle at the front but at half way of the water column. The first fish fatigued after swimming 2 h and 10 min, after swimming 10 min at 0.9 m s^–1^. The last fish fatigued after swimming 3 h and 16 min, after swimming 16 min at 1.2 m s^–1^. Data was normally distributed. Ucrit was determined at 101.5 ± 9.4 cm s^–1^ or 9.1 ± 0.7 SL s^–1^. Also for seabream, the size effect on swimming performance was removed by determination of RUcrit (Figure 3B). The linear relation between Ucrit and SL was *y* = 6.3541*x*+30.622 with *x* = SL (cm) and *y* = Ucrit in cm s^–1^.

### Experiment 2: Starvation and Refeeding

**FIGURE 4:**
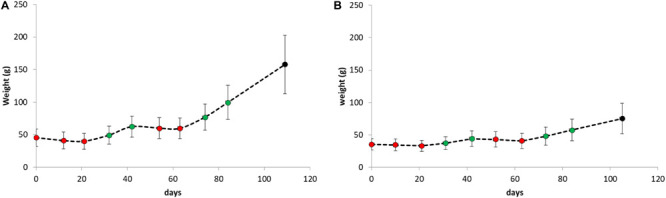
Body weight as weekly determined during the period of the replicate starvation and refeeding experiments of **(A)** Atlantic salmon and **(B)** Gilthead seabream. The red dots represent measurements during starvation periods and the green dots during refeeding periods.

Average weight gain and weight loss during the period of the replicate starvation and refeeding experiments is shown in Figure 4. For both species, weight gain and weight loss during both periods were highly significant (paired student *t*-tests *P* < 0.001).

#### Atlantic Salmon

The average feed intake (g dm d^–1^) for salmon during the first refeeding period was 0.63 ± 0.13 g dm d^–1^ (Table 1). The salmon with the lowest feed intake ate 0.33 g dm d^–1^, the salmon with the highest feed intake 0.93 g dm d^–1^, a nearly threefold difference of 0.60 g dm d^–1^. The average daily weight gain (ADG) for salmon during the first refeeding period was 1.07 ± 0.04 g d^–1^. The salmon with the lowest ADG grew 0.50 g d^–1^, the salmon with the highest ADG grew 1.67 g d^–1^, which is more than 200% faster. The average daily weight loss (ADL) for salmon during the first starvation period was 0.22 ± 0.30 g d^–1^. The formula for the predicted feed intake, based on the mean metabolic weight (MMW) and ADG for period 1, was *Y* = 0.0000445 + (0.0004373 × MMW1) + (0.5047684 × ADG1). The intercept was significantly different from zero (*P* < 0.05). The average feed conversion ratio on dry matter (FCR_DM_) for salmon during the first refeeding period was 0.59 ± 0.03, with 0.54 as the lowest and 0.66 as the highest value, a difference of 0.12.

**TABLE 1:** Starvation-refeeding parameters during period 1 and 2 for Atlantic salmon and Gilthead seabream.

	**Atlantic salmon**		**Gilthead seabream**	
	**Period 1**	**Period 2**	**Period 1**	**Period 2**
BWstart (g)	39.9 ± 12.1	59.7 ± 16.0	33.1 ± 8.5	40.6 ± 11.5
ADG (g d^–1^)	1.07 ± 0.04	1.90 ± 0.55	0.53 ± 0.21	0.83 ± 0.31
ADL (g d^–1^)	0.22 ± 0.30	0.13 ± 0.05	0.11 ± 0.04	0.17 ± 0.05
FI (g dm d^–1^)	0.63 ± 0.13	1.17 ± 0.31	0.92 ± 0.26	1.92 ± 0.57
FCR_DM_	0.59 ± 0.03	0.62 ± 0.03	1.96 ± 0.98	2.57 ± 0.98

*See text for explanation parameter abbreviations.*

The average feed intake (g dm d^–1^) for salmon during the second refeeding period was 1.17 ± 0.31 g dm d^–1^. The salmon with the lowest feed intake ate 0.62 g dm d^–1^, the salmon with the highest feed intake 1.91 g dm d^–1^, a difference of 1.29 g dm d^–1^. ADG for the salmon during the second refeeding period was 1.90 ± 0.55 g d^–1^. The salmon with the lowest ADG grew 0.89 g d^–1^, the salmon with the highest ADG grew 3.17 g d^–1^, which is more than 250% faster. ADL for salmon during the second starvation period was 0.13 ± 0.05 g d^–1^. The linear equation for the predicted feed intake, based on the MMW and ADG in period 2, was *Y* = 0.0000774 + (0.0003225 × MMW2) + (0.5514979 × ADG2). The intercept was significantly different from zero (*P* < 0.05). Average feed conversion rate on dry matter FCR_DM_ for salmon during the second refeeding period was 0.62 ± 0.03, with 0.58 as the lowest and 0.70 as the highest value, a difference of 0.12.

#### Gilthead Seabream

The average feed intake (g dm d^–1^) for seabream during the first refeeding period (period 1) was 0.92 ± 0.26 g dm d^–1^ (Table 1). The seabream with the lowest feed intake ate 0.45 g dm d^–1^, the seabream with the highest feed intake 1.39 g dm d^–1^, a difference of 0.94 g dm d^–1^. The average daily weight gain (ADG) in g d^–1^ for the seabream during the first refeeding period was 0.53 ± 0.21 g d^–1^. The seabream with the lowest ADG grew 0.16 g d^–1^, the seabream with the highest ADG grew 1.04 g d^–1^, which is 550% faster. The average daily weight loss (ADL) for seabream during the first starvation period was 0.11 ± 0.04 g d^–1^.

The linear equation for the predicted feed intake based on the MMW and ADG for period 1 is *Y* = 0.0000966 + (0.0073024 × MMW1) + (0.3536915 × ADG1). The intercept was not significantly different from zero (*P* > 0.05). Therefore, the final formula was *Y* = (0.0073024 × MMW1) + (0.3536915 × ADG1). The average feed conversion rate on dry matter FCR_DM_ for seabream during the first refeeding period (period 1) was 1.96 ± 0.98, with 1.26 as the lowest and 6.14 as the highest value, a difference of 4.88.

The average feed intake (g dm d^–1^) for seabream during the second refeeding period (period 2) was 1.92 ± 0.57 g dm d^–1^. The seabream with the lowest feed intake ate 0.72 g dm d^–1^, the seabream with the highest feed intake 2.72 g dm d^–1^, a difference of 2.00 g dm d^–1^. The average daily weight gain (ADG) in g d^–1^ for the seabream during the second refeeding period was 0.83 ± 0.31 g d^–1^. The seabream with the lowest ADG grew 0.14 g d^–1^, the seabream with the highest ADG grew 1.59 g d^–1^, which is 10 times faster. ADL for seabream during the second starvation period was 0.17 ± 0.05 g d^–1^.

The linear equation for the predicted feed intake based on the MMW and ADG for period 2 was *Y* = 0.0003325 + (0.0090532 × MMW2) + (0.6362367 × ADG2). The intercept was not significantly different from zero (*P* > 0.05). Therefore, the final formula is *Y* = (0.0090532 × MMW2) + (0.6362367 × ADG2). The average feed conversion rate on dry matter FCR_DM_ for seabream during the second refeeding period (period 2) was 2.57 ± 0.98, with 1.26 as the lowest and 6.14 as the highest value, a difference of 4.88.

### Experiment 3: Swimming Respirometry

#### Atlantic Salmon

Three fish fatigued during swimming trials at a swimming speed of 1 m s^–1^ (U_crit_ = 0.90 ± 0.02 m s^–1^). All other fish fully completed swimming trials without showing any signs of fatigue. A summary of the results of respirometry is given in Table 2. Uopt averaged 0.78 ± 0.01 m s^–1^ or 3.77 ± 0.10 SL s^–1^. COTmin was on average 153 ± 7 mg O_2_ kg^–1^ km^–1^ and MO_2_rest was 5.37 ± 0.44 mg O_2_ kg^–1^ min^–1^.

**TABLE 2:** Body weight, standard length and swimming performance parameters (average ± standard deviation) of the experimental fish (AS, Atlantic salmon; GS, Gilthead seabream) in the swim-tunnels.

	**BW**	**SL**	**Uopt**	**Uopt**	**COTmin**	**COTmin**	**MO_2_rest**	**MO_2_rest**
	**(g)**	**(cm)**	**(m s^–1^)**	**(SL s^–1^)**	**(mg O_2_ km^–1^)**	**(mg O_2_ kg^–1^ km^–1^)**	**(mg O_2_ min^–1^)**	**(mg O_2_ kg^–1^ min^–1^)**
AS	135 ± 40	21.0 ± 2.2	0.78 ± 0.08	3.77 ± 0.57	20.2 ± 6.8	153 ± 39	0.73 ± 0.33	5.4 ± 2.5
GS	68 ± 21	13.6 ± 1.4	0.61 ± 0.12	4.51 ± 0.99	16.1 ± 8.1	232 ± 88	0.76 ± 0.32	11.4 ± 3.7

*See text for explanation parameter abbreviations.*

#### Gilthead Seabream

Twenty seven fish fatigued during swimming trials at a swimming speed of 1 m s^–1^ (Ucrit = 0.91 ± 0.01 m s^–1^). Only six fish remained swimming without showing any signs of fatigue. A summary of the results is given in Table 2. Uopt averaged 0.61 ± 0.02 m s^–1^ or 4.51 ± 0.18 SL s^–1^. COTmin was on average 232 ± 16 mg O_2_ kg^–1^ km^–1^ and MO_2_rest was 11.4 ± 0.7 mg O_2_ kg^–1^ min^–1^.

**FIGURE 5:**
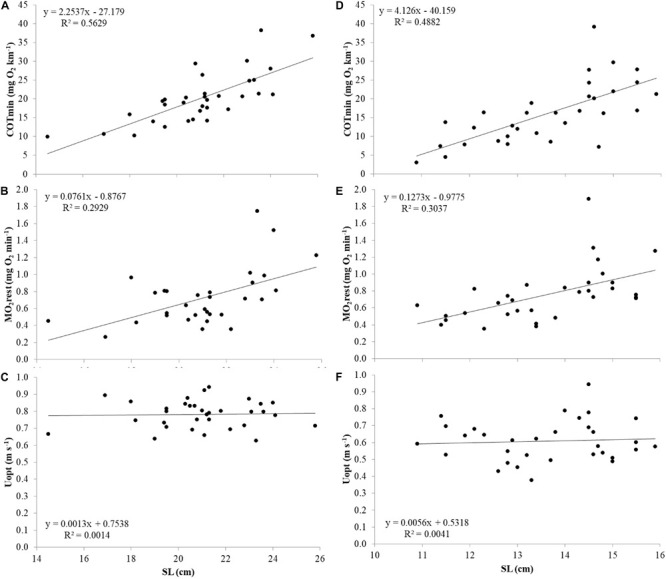
COTmin (mg O_2_ km^–1^), MO_2_ rest (mg O_2_ min^–1^), and Uopt (m s^–1^) in relation to SL from top to bottom in Atlantic salmon **(A–C)** and Gilthead seabream **(D–F)**.

For both species, the relations of COTmin (mg O_2_ km^–1^), MO_2_rest (mg O_2_ min^–1^) and Uopt (m s^–1^) with size were investigated (Figure 5). Similar to Ucrit, COTmin and MO_2_rest showed positive correlations with SL, and all three parameters showed negative correlations when corrected for size by plotting COTmin (mg O_2_ kg^–1^ km^–1^), MO_2_rest (mg O_2_ kg^–1^ min^–1^) and Uopt (SL s^–1^) vs. SL. Therefore, also for these parameters, further analysis was performed with residual values.

### Dissection

Atlantic salmon was dissected after 108 days at a body weight of 157.8 ± 44.8 g. Gilthead seabream was dissected after 105 days at a body weight of 74.9 ± 24.0 g. Measurements of dissection and body composition parameters for both species, calculated as relative to body size, are provided in Table 3.

**TABLE 3:** Atlantic salmon (AS) and Gilthead seabream (GS) carcass traits.

	**AS**	**GS**
BW (g)	157.844.8	74.924.0
Ash (%)	2.260.11	4.590.38
Cal (mJ kg^–1^)	7.480.33	7.950.78
Fat (%)	9.120.95	10.42.1
Moist (%)	70.00.9	67.31.7
Prot (%)	18.80.4	18.10.3
FATi index	2.490.90	1.680.80
Wliver index	1.030.31	1.730.28
Woes index	0.530.11	
Loes index	16.72.3	
Wstom index	0.330.05	
Lstom index	11.91.8	
Wpc index	2.080.39	
Lpc index	19.32.4	
Wmedint index	0.420.09	
Lmedint index	41.26.0	
Lfilet index	45.42.4	
Wfilet index	52.42.7	42.82.34
Wcarcass index	36.72.8	44.62.13
BWdegutted index		88.61.0
Wvisceraindex		0.280.10
Woes-stom-pcindex		0.970.23
Wintindex		1.910.31
Lintindex		10329
Hct (%)		32.64.8

*Average (AV) and standard deviation (SD) of body weight (BW, g); body composition (Ash, Fat, Moist, and Prot) in % of bodyweight and energy content (Cal) in MJ kg^–1^; indices (% of BW or TL) of visceral fat (FATi), liver weight (Wliver), osophagus weight (Woes) and length (Loes), stomach weight (Wstom) and length (Lstom), posterior intestine weight (Wpc) and length (Lpc), median intestine weight (Wmedint) and length (Lmedint), filet length (Lfilet) and weight (Wfilet), carcass weight (Wcarcass), body weight degutted (BWdegutted), viscera weight (Wviscera), osophagus-stomach-posterior intestine weight (Woes-stom-pc), intestine weight (Wint) and intestine length (Lint); and haematocrit (Hct).*

### Correlations

#### Atlantic Salmon

Body weight at the start of the starvation-refeeding trials (BWstart) was positively correlated with ADG (*R* = 0.700 and 0.757, for both periods *P* < 0.001), and negatively correlated with FCR_DM_2 (*R* = −0.343 at *P* = 0.047; Supplementary Table S1).

When swimming performance in absolute terms was considered (in cm s^–1^), large fish swam faster (Figure 3). When considering swimming in SL s^–1^, large fish swam slower and when the RUcrit is considered, the relation with size had disappeared.

The correlations between Ucrit in absolute terms (m s^–1^) and weight gains (ADG1 and 2) were positive but not significant. RUcrit did not show any significant correlation with growth but RUcrit was positively correlated with RFI2 (*R* = 0.361; *P* = 0.036). RUcrit was negatively correlated with FATindex (*R* = −0.362, *P* = 0.035).

#### Gilthead Seabream

Similar to salmon, BWstart was positively correlated with ADG (for both periods *R* = 0.756 and 0.776 at *P* < 0.001) and negatively correlated with FCR_DM_ (for both periods *R* = −0.382 and *P* < 0.05; Supplementary Table S2).

Also for seabream, large fish swam faster (Figure 3) and the correlation of RUcrit with size was not significantly different from zero.

**FIGURE 6:**
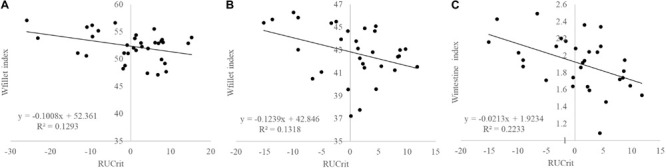
Correlations between RUcrit and **(A)** Wfilet index in Atlantic salmon (*P* = 0.125), **(B)** and Gilthead seabream (*P* = 0.034), and **(C)**, and Wintestine index in Gilthead seabream (*P* = 0.008).

Strong positive correlations existed between Ucrit in absolute terms (m s^–1^) and weight gains in seabream during the refeeding periods (ADG1: *R* = 0.494, *P* = 0.003; ADG2: *R* = 0.478, *P* = 0.005) as well as negative correlations with weight losses during starvation periods (ADL1: *R* = −0.360, *P* = 0.039; ADL2: *R* = −0.499, *P* = 0.003). RUcrit did not show any significant correlation with growth and RFI. RUcrit was negatively correlated with Wfiletindex (*R* = −0.383; *P* = 0.034; Figure 6) and Wintindex (*R* = −0.467; *P* = 0.008; Figure 6).

### Prediction of Traits

#### Atlantic Salmon

Residual COTmin had a significant predictive effect on the traits ADG in both periods and on FCR_DM_ in period 1 (Table 4). BWstart was included as a covariable in all models and showed a significant effect on all traits except on RFI in both periods and FCR_DM_ in period 1. ADG was included in the model for FI and found highly significant (Table 4). The other traits RUcrit, RUopt, and RMO_2_ did not show predictive value for the production parameters. Feed intake (FI) was found to be very well predicted by just the two covariables BWstart and ADG. For period 1 the model with BWstart and ADG explained 96% (*R*^2^) and for period 2 this was 99% of the variation in FI.

**TABLE 4:** Estimated effects of response variables on production traits in Atlantic salmon (AS) and Gilthead seabream (GS).

	**BWstart**	**ADG**	**RUcrit**	**RUopt**	**RCOTmin**	**RMO_2_rest**
**AS**						
**Period 1**						
ADG	1.610E−5*	–	−1.210E−6	1.110E−4	−1.110E−5*	−3.510E−6
FT	3.610E−7*	5.110E−1*	2.610E−7	1.510E−5	4.010E−7	−3.810E−6
FCR	−6.110E−4	–	2.510E−4	−1.510E−2	2.010E−3*	8.010E−3
RF1	1.810E−5	–	1.910E−4	1.210E−2	1.010E−3	5.910E−3
**Period 2**						
ADG	2.810E−5*	–	−3.510E−6	−6.610E−5	−2.610E−5**	−3.010E−5
FT	7.010E−7*	5.510E−1*	−4.610E−7	4.810E−6	−7.810E−8	−6.310E−6
FCR	−8.210E−4*	–	−5.510E−4	−3.210E−2	1.010E−3	5.010E−3
RF1	2.810E−5	–	9.710E−4	2.010E−2	−6.510E−5	−8.510E−4
**GS**												
**Period 1**						
ADG	1.810E−5*		2.810E−6	−7.210E−5	−5.510E−6	−2.010E−5
Fl	1.110E−5*	5.510E−1*	2.610E−6	−9.510E−6	−1.310E−5**	−1.110E−4
FCR	−2.510E−2		1.010E−2	8.710E−1	−1.310E−2	−6.810E−2
RF1	−8.510E−5		2.910E−3	1.110E−2	−1.210E−2*	−1.110E−1
**Period 2**						
ADG	2.310E−5*		−2.610E−6	−3.710E−4	−9.810E−6	1.010E−4
Fl	1.210E−5*	9.710E−1*	4.110E−6	−8.210E−6	−2.110E−5	−3.110E−4
FCR	−3.410E−2*		1.410E−3	1.2	−4.410E−3	−3.610E−1
RF1	−5.010E−4		3.610E−3	−1.010E−1	2.010E−2^∧^	−2.710E−1

**P < 0.01; *P < 0.05; ^∧^*P* < 0.10. See text for abbreviations.

For carcass traits, RUcrit had a significant predictive effect on Wfilet (*P* < 0.05), and a possible predictive effect on Lpc, Wmedint, Lmedint, and Wcarcass (*P* < 0.10).

#### Gilthead Seabream

Residual COTmin has a significant predictive effect on both FI and RFI in the first testing period and in the second period there is a possible effect (*P* < 0.10) on RFI. BWstart was included as a covariable in all models and showed a significant effect on all traits except on RFI in both periods and FCR_DM_ in period 1. ADG was included in the model for FI and found highly significant (Table 4). The other predictors RUcrit, RUopt, and RMO_2_ did not show predictive value for the production parameters, the same result as seen in the data on salmon. In contrast to salmon, RCOTmin showed a significant prediction for FI, on top of the significant effects of BWstart and ADG. In seabream BWstart and ADG are much less predictive of FI, with *R*^2^ values of 59% and 58% for periods 1 and 2, respectively, with only BWstart and ADG in the model.

For carcass traits, RUcrit had a significant predictive effect on Wfilet index (P < 0.05). Furthermore, RUcrit had significant effects on FATi, Wint, and Wcarcass.

## Discussion

The aim of this study, was to identify accurate indicators for target traits of selective breeding for Atlantic salmon and Gilthead seabream. Specifically, the predictive value of critical swimming speed Ucrit and RUcrit, and the indicative value of respirometric parameters RUopt, RCOTmin, and RMO_2_rest, for production and carcass traits were assessed. A critical swimming speed test was designed to provide individual Ucrit values in high throughput manner. Different from earlier investigations, RUcrit values were used for this purpose as Ucrit in absolute terms (ms s^–1^) correlates with SL.

### The Ucrit Test, Ucrit and Residual Ucrit Values

Ucrit can be determined in high throughput manner in a swim gutter depending on the induced flow range that can be reached, the swimming durations at each speed and the size of the fish. In this study, a swim gutter was used in which flows could be induced of maximum 1.2 m s^–1^. A protocol was designed to characterize the Ucrit of 34 fish in 200 min for both Atlantic salmon and Gilthead seabream. When a maximal individual variation of two times standard deviation is considered, the maximum size of the fish can be calculated from the relation between Ucrit and SL being 13 cm SL for Atlantic salmon and 11 cm SL for Gilthead seabream using this experimental set-up and protocol.

Surprisingly, small seabream in this study swam faster than salmon (101.5 ± 9.4 cm s^–1^ or 9.1 ± 0.7 SL s^–1^ vs. 95.9 ± 10.3 cm s^–1^ or 7.9 ± 1.0 SL s^–1^). Sizes were similar although salmon was longer in length than seabream (12.3 ± 1.6 vs. 11.2 ± 1.0 cm) and seabream was heavier than salmon (36.5 ± 9.4 vs. 29.9 ± 9.9 g). At larger size in the swim tunnels, salmon performed better than seabream. Only three salmons fatigued and the remaining 31 fish were able to finish the trial swimming at 1.0 m s^–1^ while 27 seabream fatigued at an average Ucrit of 0.91 ± 0.01 m s^–1^. The Ucrit that we found for seabream was much higher than the 3.4 ± 0.2 BL s^–1^ that was reported by Steinhausen et al. (2010) which may be explained by the larger size of the fish in that study, the short swimming section of the respirometer that was used in comparison with the “endless” swim gutter in our study (Tudorache et al., 2007) and, particularly, by the fact that this value was determined on individual fish while in our study fish were schooling. This reasoning was supported by the fact that the results for salmon were comparable with results from other group-swimming salmon trials in a raceway-type swim gutter (Remen et al., 2016).

The Ucrit in cm s^–1^ shows clear relation with fish size: larger fish swim faster in absolute terms (Bainbridge, 1958; Brett and Glass, 1973). To correct for the size effect, the RUcrit was used in this study which is the residual variation in Ucrit not explained by size (SL): Ucrit = a + b × SL + e. As expected, RUcrit did not show any dependency on SL (Figure 3).

### Starvation and Refeeding

Over the two starvation-refeeding periods, the individually housed salmon increased 120% in weight. Both ADG and FI doubled from period 1 to period 2. As a consequence, FCR_DM_ remained similar at ∼0.6 on dry matter base which is comparable to an aquaculture situation. Seabream increased only 23% in weight and the FCR_DM_ was high at 1.96 during period 1 and increased up to 2.57 during period 2. The individual variation was about 30-fold higher than in salmon which could be a result of selective breeding efforts for growth in this strain of salmon, which have not been undertaken to this extent for the experimental seabream. Another important explanation for the high FCR_DM_ in seabream may originate from the fact that seabream crunch the pellets. Crunching may prevent the fish from efficient uptake and has important consequences for maintaining water quality in RAS (also Dosdat et al., 1996). An important question would be if there is genetic variation for crunching behavior (e.g., heredity of crunching). Crunching could then be targeted by selective breeding.

#### Swim Tunnel Respirometry

Few reported data exist on the swimming capacities of juvenile seabream so the contribution of this study to this knowledge base is important. Swimming capacities of salmonids have been studied intensively.

The experimental seabream in this study had an average absolute Uopt of 0.61 m s^–1^ which corresponded to a high average relative Uopt of 4.51 SL s^–1^ because of their short, deep body shape. Seabream can swim at this speed continuously for a long period as was shown in a 24-days swim-training trial (Palstra et al., 2015). Steinhausen et al. (2010) reported an Uopt of 2.3 BL s^–1^ for larger farmed seabream (0.201 ± 0.010 kg and 22.8 ± 0.53 cm). These results are in line with conclusions for juvenile yellowtail kingfish that show that the relative Uopt for small fish is high but drops rapidly with increasing size (Palstra et al., 2015). The data for salmon in this study emphasize how the relative Uopt can be much higher for smaller fish than the generally applied swimming speeds of 0.8 to 1.5 BL s^–1^ in salmon aquaculture which are only appropriate for the large adults (reviewed by Davison and Herbert, 2013).

Oxygen consumption rates for the seabream in this study were higher as compared to the fish in the study of Steinhausen et al. (2010), 1.6-fold higher for metabolic rate at Uopt and fivefold higher for the standard metabolic rate. Results are in line as the fish in our study were smaller and oxygen consumption (in mg O_2_ kg^–1^ h^–1^) generally shows a logarithmic decrease with size. Similarly, oxygen consumption rates for salmon were in line with to those reported in literature for juvenile salmon of different sizes (Enders et al., 2003; Beauregard et al., 2013; Oligny-Hébert et al., 2015; Alexandre and Palstra, 2017).

### The Predictive Value of Ucrit

In general, a trade-off is considered between growth rates and swimming performance in absolute terms, i.e., fast swimmers have lower (compensatory) growth rates (Farrell et al., 1997; Gregory and Wood, 1998, 1999; Killen et al., 2014). In these studies, swimming performance was assessed during periods of growth and not before them in order to test the predictive value of swimming traits as was done in our study. We found however strong positive correlations between Ucrit in m s^–1^ and weight gains in seabream during refeeding periods (as well as negative correlations with weight losses during starvation periods). In Atlantic salmon, the correlations between Ucrit in m s^–1^ and weight gains were also positive but not significant. Our results are comparable with those reported for European seabass by Vandeputte et al. (2016). Also for seabass, Ucrit in m s^–1^ was positively correlated with body weight, and negatively when it was expressed in relative terms (in Body Lengths s^–1^). Interestingly, heritability was high when expressed in Body Lengths s^–1^ (h2 = 0.55 ± 0.08), but this appeared to be a size effect. We did not find any significant correlation between RUcrit with growth, but we did find strong correlations between BWstart and growth confirming that size alone can predict growth in Atlantic salmon and Gilthead seabream.

From the positive correlation between RUcrit and RFI2 in Atlantic salmon, it can be concluded that the critical swimming speed test may predict the feed intake later in life. Fast swimming then correlates with higher feed intake. From the negative correlation between RUcrit and FATiindex, it can be concluded that the critical swimming speed test may predict the amount of intestinal fat at slaughter size. Fast swimming then correlates with less intestinal fat. Although functional interpretation of correlations requires cautiousness, it appears that fast-swimming fish may be more active fish which have higher feed intake which does not necessarily result in better growth performance or filet yield, nor intestinal fat deposition. Fast swimming Atlantic salmon may thus not be targeted for selection unless fast swimming relates to robustness traits such as stress coping and disease resistance. Physiotyping of fast and slow swimmers by stress and immune challenge tests could provide such data. The findings of Castro et al. (2013) provide support for a relation between the inherent swimming performance and disease resistance in Atlantic salmon.

Surprisingly, we found a negative correlation between RUcrit and Wfiletindex in both Atlantic salmon and Gilthead seabream, from which it can be concluded that the critical swimming speed test could predict filet yield. Good swimming performance correlates with less filet yield indicating that fast-swimming fish may be the more slender fish. So the slow swimming and not the fast swimming phenotype may be targeted for selection unless the more slender fish have a body shape that is preferred by the customer. Seabream and other Mediterranean fish are often sold as whole body fish and higher resemblance to wild-type fish may be preferred (also Fragkoulis et al., 2017).

Atlantic salmon is an athletic fish, known for its sustained subcarangiform swimming movements during long-term migrations in the Atlantic Ocean but also during fast sprints in the rapid freshwater streams. Gilthead seabream is a high-bodied fish built to maneuver in the tides that performs carangiform swimming in schools at lower speeds and faster burst-and-glide sprinting. Although both species have such different life styles and occupy very different niches, they share the negative correlation between fast swimming as young juveniles and lower filet yield later in life. This correlation may indicate that slenderness decreasing drag and promoting fast swimming follows general hydrodynamics and can be also found for other fishes. The slender fast swimming phenotype is furthermore characterized by less intestinal fat, less intestine tissue and heavier bone structure (given the negative RUcrit effects on intestinal fat and intestine, and positive effect on carcass weight; Table 5). When slender fish are more active fish, then quantitative differences in the muscle can be expected as well. High activity may have similar effects as reflected by exercise training leading to white skeletal muscle hypertrophy by enlarged fibers (salmon: Totland et al., 1987; seabream: Ibarz et al., 2011) and filet hardness, probably by cross-linking collagen fibrils (trout: Rasmussen et al., 2013).

**TABLE 5:** Estimated effects of residual critical swimming speed (RUcrit) on carcass traits in Atlantic salmon (AS) and Gilthead seabream (GS).

	**BW**	**RUcrit**
**AS**		
Ash	–0.001	–0.001
Cal	ns	–0.004
Fat	ns	–0.012
Moist	ns	0.016
Prot	ns	0.004
FATi	0.017	–0.023
Wliver	0.01	0.001
Woes	0.005	0.002
Loes	0.009	–0.005
Wstom	0.003	–0.001
Lstom	0.005	–0.006
Wpc	0.027	0.013
Lpc	0.011	0.017^∧^
Wmedint	0.004	0.004^∧^
Lmedint	0.018	0.039^∧^
Wfilet	0.525	−0.189*
Lfilet	0.047	–0.003
Wcarcass	0.378	0.148^∧^
GS		
Ash	–0.009	0.005
Cal	0.023	–0.004
Fat	0.064	0.011
Moist	–0.046	0.019
Prot	ns	0.006
FATi	0.029	0.028*
Wliver	0.019	–0.002
Wfilet	0.467	−0.076*
Wcarcass	0.417	0.071*
BWdegutted	0.887	–0.003
Wviscera	0.004	–0.002
Woes-stom-pc	0.008	–0.001
Wint	0.018	−0.018**
Lint	0.057	–0.108
Hct	0.072	0.104

Linear regression was tested with BW as cofactor only when significant (ns, not significant). Wfilet was negatively related to RUcrit in salmon and in bream. For seabream, additionally, significant relations existed between RUcrit and FATi. Wint and Wcarcass. For the latter two traits, trends existed in salmon. One outlier (>2 SD) was removed for Wliver in salmon. **P < 0.01; *P < 0.05; ^∧^*P* < 0.10. See text for abbreviations.

### The Indicative Value of Minimal Cost of Transport for Growth Potential

In this study, we have shown that RCOTmin had predictive value for response variables such as ADG, FI, FCR_DM_ and RFI. In our model, a higher BWstart is a predictor of higher feed intake and fast growth as well as lower FCR_DM_ which could be expected from an exponential growth curve. An important research question would be why these fish were already bigger at the start of the experiments, therewith a topic for future research. As for the predictive value of RCOTmin on feed intake, the *R*^2^ values were already very high for Atlantic salmon (0.96 and 0.99 in period 1 and 2) when BWstart and ADG were included. Very little variation was left in this case for RCOTmin to provide significant added value. Probably, the *R*^2^ values were so high because these salmons were already a product of selective breeding for growth. For seabream this was not the case and *R*^2^ values from a model with BWstart and ADG were much lower. Here RCOTmin provided significant additional predictive value to the model. Determining the RCOTmin could be an alternative to measuring individual FI in fish, which is very difficult to do (de Verdal et al., 2017). However, to measure RCOTmin requires individual oxygen consumption measurements while swimming at Uopt, which in the current experiments is also time consuming and low throughput. To measure RCOTmin in high throughput manner would ask for the development of oxygen sensors.

## Conclusion

Ucrit can be determined in relatively high throughput manner and could be used as a predictor for other target traits for selective breeding of Atlantic salmon and Gilthead seabream, such as RFI and filet-%. However, the RUcrit should be considered in order to remove the size dependency of swimming performance. The RUcrit predicted filet yield in both species. The minimal COT, the oxygen consumption when swimming at Uopt, adds predictive value to the seabream model for feed intake.

## Data Availability Statement

The raw data supporting the conclusions of this article will be made available by the authors, without undue reservation.

## Ethics Statement

The studies involving animals were reviewed and approved by the animal experimental committee of Wageningen Research (DEC nr. 2014064).

## Author Contributions

AP and JK contributed to the conceptualization and methodology of the study. AP, JK, and TB contributed to the investigation. AP, JK, JB, and HK contributed to the formal analysis. AP, JK, and JB contributed to the preparation and writing of the original draft. AP, JK, JB, and HK contributed to the review and editing of the writing. All authors read and approved the final manuscript.

## Conflict of Interest

The authors declare that the research was conducted in the absence of any commercial or financial relationships that could be construed as a potential conflict of interest.
